# Prenatal and childhood exposure to smoking and retinal nerve fibre layer thickness: A meta‐analysis of three independent birth cohorts

**DOI:** 10.1111/aos.70109

**Published:** 2026-03-07

**Authors:** Linna Zhu, Inger Christine Munch, Samantha Sze‐Yee Lee, David A. Mackey, Tamo Sultan, Klaus Bønnelykke, Bo Chawes, Michael Larsen, Nicklas Brustad

**Affiliations:** ^1^ Department of Ophthalmology Rigshospitalet Glostrup Denmark; ^2^ Department of Clinical Medicine, Faculty of Health and Medical Sciences University of Copenhagen Copenhagen Denmark; ^3^ Centre for Clinical Research and Prevention Bispebjerg and Frederiksberg Hospital Frederiksberg Denmark; ^4^ The University of Western Australia, Centre for Ophthalmology and Visual Science (Incorporating the Lions Eye Institute) Nedlands Western Australia Australia; ^5^ Copenhagen Prospective Studies on Asthma in Childhood Copenhagen University Hospital, Herlev‐Gentofte Denmark

**Keywords:** optic nerve, optical coherence tomography, retinal nerve fibre layer, smoking, tobacco

## Abstract

**Purpose:**

To examine retinal nerve fibre layer (RNFL) characteristics in relation to prenatal and postnatal smoking exposure in three independent birth cohorts: two Danish and one Australian cohort.

**Methods:**

A combined meta‐analysis of peripapillary retinal nerve fibre thickness in the Copenhagen Prospective Studies on Asthma in Childhood 2000 cohort (COPSAC2000), the Copenhagen Child Cohort 2000 (CCC2000), and Generation 2 of the Raine Study, utilizing optical coherence tomography.

**Results:**

Compared with participants not exposed to smoking (COPSAC2000 = 209, CCC2000 = 1008, Raine Study Gen2 = 991), 35 COPSAC2000 participants exposed to smoking throughout pregnancy had the most prominent deficits in peripapillary RNFL thickness in the inferior (−10.4% (−16.7; −4.2%), *p* = 0.001) and nasal (−10.0% (−18.3; −1.7%), *p* = 0.02) sectors, while 221 CCC2000 participants exposed to smoking had the most prominent deficit in the inferonasal sector (−8.4% (−11.3; −5.4%), *p* < 0.001), and 112 Raine Study Gen2 participants exposed to smoking (more than 10 cigarettes per day) had the most prominent deficit in the inferonasal sector (−8.9% (−13.0; −4.8%), *p* < 0.001). In a combined meta‐analysis of the three cohorts, deficits in both the global and sectoral (superior, nasal, inferior and temporal) RNFL were observed from exposure to prenatal smoking.

**Conclusions:**

Prenatal smoking was associated with deficits in the peripapillary RNFL both globally and sectorally in combined meta‐analyses of the three cohorts, which suggest an overall adverse effect of early life smoking exposure on later RNFL status.

## 1 INTRODUCTION

The retinal nerve fibre layer (RNFL) is composed of ganglion cell axons that converge on the optic nerve head. (Shi et al., 2019) Recent population‐based cohort studies have shown that RNFL deficits are associated with lower birth weight, longer ocular axial length, exposure to tobacco smoke and lower physical working capacity (Ashina et al., 2017; Lee, Mackey, et al., 2021; Lee, McVeigh, et al., 2021; Pueyo et al., 2011; Zhu et al., 2023). The prevalence of daily smokers among people aged 15 years or older in Europe in 2019 ranged from 6.4% to 28.7%. (Eurostat, 2019). Despite the high prevalence of smoking, little is known about how tobacco smoking affects the ganglion cells of the retina, their RNFL axons, and their continuation in the optic nerve. Prior studies have shown that different optic neuropathies have a predilection for specific sections of the retinal ganglion cell layer. Thus, autosomal‐dominant optic atrophy (ADOA) has a peripapillary nerve fibre layer deficit in its early stages that is most prominent in the inferotemporal peripapillary RNFL sector (Park & Hwang, 2015; Rönnbäck et al., 2013). This is different from the predilection of glaucomatous nerve fibre layer deficits for the inferior peripapillary RNFL sector (Bowd et al., 2000; Kwon et al., 2009; Larrosa et al., 2015) and that of Leber hereditary optic neuropathy for the papillomacular bundle (Park & Hwang, 2015; Wang et al., 2021).

The present study examined the topographical distribution of RNFL deficits related to prenatal and postnatal tobacco exposure in three cohorts to try to determine if it is also unevenly distributed and, if so, in what type of pattern.

## 2 MATERIALS AND METHODS

The study combined data from three cohorts that were examined using comparable optical coherence tomography (OCT) acquisition techniques on platforms that provide RNFL thickness analyses divided into four equally large quadrants (superior, nasal, inferior, temporal (COPSAC)) or six sectors that differ from the former in that upper and the lower quadrants are divided in two along a vertical meridian (CCC2000 and the Raine Study Gen2). Summarization of outcomes were made by identification of the most severely affected sector or sectors, the latter if there were or more that were affected to nearly the same degree.

### 2.1 COPSAC2000

#### 2.1.1 Study participants

The Copenhagen Prospective Studies on Asthma in Childhood 2000 (COPSAC_2000_) is a single‐centre clinical, prospective, mother–child cohort study of 411 children born in year 2000 in greater Copenhagen, Denmark of mothers with a history of asthma who were recruited during pregnancy from the Danish National Birth. (Bisgaard, 2004). Eye examinations were part of the 18‐year follow‐up. (Mølbæk‐Engbjerg et al., 2024) Of the 411 children, 269 were included in the analyses. The left eye was the default eye used in data analyses, except in 53 cases where scans were deemed to be of substandard quality, hence data from the right eye were used instead. One participant was undergoing current work‐up for retinitis pigmentosa and was excluded from the study (Zhu et al., 2023).

#### 2.1.2 Smoking exposure

Maternal tobacco smoking was registered 2 weeks after giving birth and was categorized as (1) not smoking during pregnancy or (2) smoking during pregnancy. If the mothers smoked during pregnancy, they were asked how many cigarettes they smoked per week and in which trimesters they smoked. Passive smoking during childhood (environmental tobacco smoke during childhood) was categorized as (1) the child having been exposed to passive smoking at home during the first 7 years of life or (2) the child not having been exposed to passive smoking at home during the first 7 years of life. Smoking at 18 years of age was categorized as (1) smoking at least 1 cigarette during the last 4 weeks or (2) not having smoked during the last 4 weeks (Zhu et al., 2023).

#### 2.1.3 Eye examination

RNFL was measured using OCT (Cirrus HD‐OCT, Carl Zeiss Meditech, Jena, Germany) in the form of 6 mm × 6 mm optic disc cube consisting of 200 horizontal line scans. The total average peripapillary RNFL thickness and the average of the four individual sectors (superior, nasal, inferior and temporal) were measured automatically using the built‐in software. Scans were only accepted if they had been fully completed, were well centred, had a signal strength of at least 6 on a scale up to 10 and had no blinking or motion artefacts (Zhu et al., 2023).

### 2.2 Copenhagen child cohort 2000 (CCC2000)

#### 2.2.1 Study participants

The CCC2000 study is a population‐based cohort study comprising all children born in greater Copenhagen, Denmark, in year 2000 and included eye follow‐up examinations of children when they were aged 11 or 12 years. 1229 children had available eye data and data on maternal smoking throughout pregnancy. Of these, 1008 non‐exposed children and 221 children exposed to smoking throughout pregnancy. Excluded participants had congenital malformations, prior eye trauma or examinations of substandard quality (Ashina et al., 2017).

#### 2.2.2 Smoking exposure

Information about self‐reported maternal smoking during pregnancy was retrieved from the Danish Medical Birth Registry. Maternal smoking was categorized as (1) non‐smoking from before conception, (2) smoking at conception but stopped during pregnancy and (3) continued smoking throughout pregnancy (Ashina et al., 2017).

#### 2.2.3 Eye examination

RNFL thickness was measured using OCT (Spectralis HRA + OCT, Heidelberg Engineering). Scanning procedures included a standard 12°, disc‐centred circular peripapillary scan in high resolution, averaging more than 25 B scans. RNFL thickness was measured automatically using the manufacturer's software (Heidelberg Eye Explorer, version 1.6.1.0; Heidelberg Engineering), and the measurements were reported as mean circumpapillary RNFL thickness and sectorial peripapillary RNFL thickness in these six sectors: superonasal, nasal, inferonasal, inferotemporal, temporal and superotemporal. Scans with a quality of less than 25 dB were excluded (Ashina et al., 2017).

### 2.3 The Raine study

#### 2.3.1 Study participants

The Raine study is a longitudinal study in Western Australia that has been following a birth cohort since their prenatal period. The Raine Study started with 2900 pregnant women who were recruited at 16‐ to 18‐weeks' gestation, and a total of 2868 children (Generation 2) were born to these mothers (Generation 1) in 1989 to 1992. At the Generation 2 (Gen 2) 20‐year follow‐up, 1344 participants attended a comprehensive eye examination. Of these, 1260 participants' eyes were analysed where 991 were not exposed to maternal smoking, and 269 were exposed to maternal smoking throughout pregnancy—of these 112 participants had mothers smoking >10 cigarettes per day. Both eyes were used, and generalized estimating equations were used to account for the correlation between the two eyes. Excluded participants had eye pathology or eye examinations of unacceptable quality (Lee, Mackey, et al., 2021).

#### 2.3.2 Smoking exposure

At 18‐ and 34‐weeks' gestation, information on mothers' smoking habits was collected prospectively using a self‐administered questionnaire, including the quantity per week (number of cigarettes, collected as categorical data) and if they ceased these habits during their pregnancy. At the Gen2 20‐year follow‐up, participants (then 19–22 years of age) completed a questionnaire that collected information on their current smoking status, including cigarettes smoked per day and age at which they started smoking. The number of pack‐years for each participant was defined as the number of packs (of 20 cigarettes) smoked per day multiplied by the number of years they had been smoking (Lee, Mackey, et al., 2021).

#### 2.3.3 Eye examination

Participants underwent spectral‐domain OCT (SD‐OCT) imaging (Spectralis HRA+OCT, Heidelberg Engineering, Heidelberg, Germany). A circular scan of 3.5 mm in diameter centred on the optic nerve head was obtained, and the total average peripapillary RNFL thickness and the thickness of the six peripapillary sectors were measured automatically using the manufacturer's software. (Lee, Mackey, et al., 2021).

### 2.4 Statistical analysis

Mean and standard deviation (SDs) were calculated for continuous variables: median and interquartile range for skewed distributions (age). Associations between RNFL and exposure variables were assessed using a general linear regression model. Sex, smoking, alcohol use and inhaled plus intranasal corticosteroid use were entered as categorical data. Age, birth weight, and axial length were entered as continuous data. Assumptions of linearity, variance homogeneity, normality of distribution of residuals underlying the statistical model and missing data were validated by visual inspection of plots. Analyses were adjusted by including relevant variables in a general linear model, and estimates are presented with 95% CIs. The mean percentual smoking‐related RNFL thickness deficit was calculated for each peripapillary RNFL sector in each of the three cohorts, and the values compared using multiple linear regression (Table 1).

**TABLE 1:** Characteristics of the study population stratified by maternal smoking throughout pregnancy.

Characteristics	No maternal smoking during pregnancy	Maternal smoking throughout pregnancy
COPSAC2000 cohort		
Number of participants, *n*	209 (78%)	35 (13%)
Age, years^a^	17.6 (0.6)	17.5 (0.8)
Boys/girls, %	53.4/46.6	57.1/42.9
Axial length, mean (SD), mm	23.6 (0.9)	23.3 (0.8)
Retinal nerve fibre layer thickness, mean (SD), μm	95.8 (9.2)	87.9 (9.7)
Best corrected visual acuity^b^, mean (SD)	1.22 (0.1)	1.16 (0.2)
CCC2000 cohort		
Number of participants, *n*	1008 (82%)	221 (18%)
Age, mean (SD), years	11.7 (0.4)	11.7 (0.4)
Boys/girls, %	47.4/52.6	48.4/51.6
Axial length, mean (SD), mm	23.2 (0.77)	23.1 (0.75)
Retinal nerve fibre layer thickness, mean (SD), μm	105 (9.4)	100 (9.6)
Best corrected visual acuity, mean (SD), no. of ETDRS letters	90.0 (2.7)	89.6 (2.6)
The Raine Study Gen2		
Number of participants, *n*	991 (78.7%)	269 (21.3%)^c^
Age, mean (SD), years	20.0 (0.4)	20.1 (0.5)
Males/females, %	46.9/53.1	45.0/55.0
Axial length, mean (SD), mm	23.7 (0.91)	23.4 (0.88)
Retinal nerve fibre layer thickness, mean (SD), μm	101.5 (13.6)	96.6 (10.1)
Best corrected visual acuity, mean (SD), logMAR	−0.06 (0.08)	−0.05 (0.08)

Abbreviation: ETDRS, Early Treatment Diabetic Retinopathy Study.

^a^ Variable presented as median and interquartile range because of skewed distribution.

^b^ Correct identification of at least three out of five letters per line (Auto Ref/Keratometer ARK 1‐s, NIDEK).

^c^ 269 mothers smoked throughout pregnancy in The Raine Study Gen2, where 112 mothers smoked >10 cigarettes/day.

Meta‐analyses were performed using cohort‐specific adjusted mean absolute differences in peripapillary RNFL thickness (μm) from the primary regression models. Pooled effects were estimated using a random‐effects model fitted by restricted maximum likelihood (REML). Study weights were assigned using inverse‐variance weighting, and statistical heterogeneity was assessed using the *I* ^2^ statistic, between‐study variance (*τ* ^2^) and Cochran's *Q* (with degrees of freedom and *p*‐value). RNFL regions were harmonized between cohorts. Nasal and temporal sectors were used as reported in each cohort. For CCC2000 and The Raine Study Gen2, superior and inferior quadrants were approximated by averaging adjacent sectors (superior = mean of superonasal and superotemporal; inferior = mean of inferonasal and inferotemporal). Since sector‐specific estimates within the same eye are not independent but correlated, standard errors for these averaged sectors were derived assuming a within‐eye correlation of *ρ* = 0.8 between the two sector estimates. The level of significance was set at a two‐sided *p* < 0.05. Statistical analyses were made using the R software package, version 4.3.1 (R Foundation for Statistical Computing, www.r‐project.org).

## 3 RESULTS

### 3.1 Prenatal smoking exposure

#### 3.1.1 COPSAC2000

Of the 269 participants included in this study (median [IQR] age 17.6 [0.6] years), 124 (46%) were boys. Mean (SD) peripapillary RNFL thickness was 94.6 μm (9.6).

The 35 (13.0%) participants whose mothers smoked throughout pregnancy had a thinner peripapillary RNFL than the offspring of non‐smoking mothers (mean relative difference −8.1%; 95% CI −12.2 to −4.1%; *p* = 0.0001) and the adjusted sector analysis found significant deficits in 3 out of 4 sectors, namely superior (−8.9%; 95% CI −15.1 to −2.8%; *p* = 0.005), nasal (−10.0%; 95% CI −18.3 to −1.7%; *p* = 0.02) and inferior (−10.4%; 95% CI −16.7 to −4.2%; *p* = 0.001), peaking at comparable magnitudes in the adjacent inferior and nasal sectors (Table 2).

**TABLE 2:** Prenatal smoking and associations with retinal nerve fibre layer thickness in young adults aged 18 years (COPSAC2000).

RNFL sector	RNFL thickness, mean (SD), μm, no smoking (*n* = 209)	Ceased smoking during pregnancy vs. no smoking, mean absolute difference (95% CI)^a^, μm (*n* = 22)	*p* Value	Smoking throughout pregnancy vs. no smoking, mean absolute difference (95% CI)^a^, μm (*n* = 35)	Smoking throughout pregnancy vs. no smoking, mean relative difference (95% CI)^a^, % (*n* = 35)	*p* Value
Global	95.8 (9.2)	−2.6 (−6.6 to 1.4)	0.21	−7.8 (−11.7 to −3.9)	−8.1 (−12.2 to −4.1)	0.0001*
Superior	111.9 (15.8)	−0.1 (−7.1 to 6.9)	0.97	−10.0 (−16.9 to −3.1)	−8.9 (−15.1 to −2.8)	0.005*
Nasal	66.3 (16.1)	−3.7 (−9.3 to 1.9)	0.19	−6.6 (−12.1 to −1.1)	−10.0 (−18.3 to −1.7)	0.02*
Inferior	112.1 (16.1)	−2.9 (−10.0 to 4.2)	0.43	−11.7 (−18.7 to −4.7)	−10.4 (−16.7 to −4.2)	0.001*
Temporal	61.1 (9.8)	−3.8 (−7.9 to 0.3)	0.07	−3.2 (−7.2 to 0.8)	−5.2 (−11.8 to 1.3)	0.12

^a^ Adjusted for age, sex, birthweight, axial length, passive smoking at home during childhood, smoking at 18 years, maternal alcohol use, alcohol use at 18 years and inhaled and/or intranasal corticosteroid use.

* Significant at two‐sided *p* < 0.05.

#### 3.1.2 CCC2000

Of the 1229 participants included in this study (mean [SD] age 11.7 [0.4] years), 644 (52.4%) were boys. Mean (SD) peripapillary RNFL thickness was 104 μm (9.6).

The 221 (18%) participants whose mothers smoked throughout pregnancy had a lower peripapillary RNFL than the offspring of non‐smoking mothers (*n* = 1008) and the adjusted analysis found significant deficits in 6 out of 6 sectors, reaching a maximum of −8.4% (−11.3 to −5.4%, *p* < 0.001) in the inferonasal sector (Table 3).

**TABLE 3:** Prenatal smoking and associations with retinal nerve fibre layer thickness in children aged 11 or 12 years (CCC2000).

RNFL sector	RNFL thickness, mean (SD), μm, no smoking exposure (*n* = 1008)	Smoking throughout pregnancy vs no smoking, mean absolute difference (95% CI)^a^, μm (*n* = 221)	Smoking throughout pregnancy vs. no smoking, mean relative difference (95% CI)^a^, % (*n* = 221)	*p‐*Value
Global	105 (9.4)	−5.7 (−7.1 to −4.3)	−5.4 (−6.8 to −4.1)	<0.0001*
Superonasal	115 (22.2)	−5.7 (−9.0 to −2.4)	−5.0 (−7.8 to −2.1)	0.008*
Nasal	81.5 (13.9)	−3.5 (−5.6 to −1.3)	−4.3 (−6.9 to −1.6)	0.002*
Inferonasal	116 (23.3)	−9.7 (−13.1 to −6.3)	−8.4 (−11.3 to −5.4)	<0.001*
Inferotemporal	152 (18.5)	−8.9 (−11.7 to −6.1)	−5.9 (−7.7 to −4.0)	<0.001*
Temporal	74.4 (10.2)	−3.7 (−5.2 to −2.1)	−5.0 (−7.0 to −2.8)	<0.001*
Superotemporal	148 (17.3)	−7.0 (−9.6 to −4.3)	−4.7 (−6.5 to −2.9)	<0.001*

*Note*: All used CCC2000 data have been published, we conducted further analyses (Ashina et al., 2017).

^a^ Adjusted for age, sex, height, weight, Tanner stage, birthweight, axial length, spherical equivalent refractive error and maternal smoking during pregnancy (Ashina et al., 2017).

* Significant at two‐sided *p* < 0.05.

#### 3.1.3 The Raine study Gen2

Of the 1260 participants included in this study (mean [SD] age 20.0 [0.4] years), 616 (51.3%) were males. Mean (SD) peripapillary RNFL thickness was 100.4 μm (13.1).

The 112 (8.9%) participants whose mothers smoked more than 10 cigarettes/day throughout pregnancy had a lower peripapillary RNFL than the offspring of non‐smoking mothers. The adjusted analysis found significant deficits in 6 out of 6 sectors, peaking at a relative magnitude of −8.9% (−13.0 to −4.8%, *p* < 0.001) in the inferonasal sector (Table 4).

**TABLE 4:** Prenatal smoking and associations with retinal nerve fibre layer thickness in young adults aged 19–22 years (The Raine Study Gen2).

RNFL sector	RNFL thickness, mean (SD), μm, no smoking exposure (*n* = 974)	Smoking throughout pregnancy >10 cigarettes/day vs. no smoking, mean absolute difference (95% CI)^a^, μm (*n* = 112)	Smoking throughout pregnancy >10 cigarettes/day vs. no smoking, mean relative difference (95% CI)^a^, % (*n* = 112)	*p‐*Value
Global	101.5 (13.7)	−6.6 (−8.7 to −4.4)	−6.5 (−8.6 to −4.3)	<0.001*
Superonasal	110.8 (22.7)	−5.9 (−10.1 to −1.7)	−5.3 (−9.1 to −1.5)	0.006*
Nasal	80.1 (20.3)	−4.1 (−6.9 to −1.2)	−5.1 (−8.6 to −1.5)	0.006*
Inferonasal	112.2 (30.7)	−10.0 (−14.6 to −5.4)	−8.9 (−13.0 to −4.8)	<0.001*
Inferotemporal	145.4 (29.2)	−9.5 (−13.1 to −5.9)	−6.5 (−9.0 to −4.1)	<0.001*
Temporal	72.2 (13.3)	−5.7 (−7.5 to −3.8)	−7.9 (−10.4 to −5.3)	<0.001*
Superotemporal	139.4 (18.9)	−9.5 (−13.5 to −5.5)	−6.8 (−9.7 to −3.9)	<0.001*

*Note*: Supplementary summary statistics data used in the current study was requested from and sent by investigators of the Raine Study (Lee, Mackey, et al., 2021).

^a^ Adjusted for sex, ethnicity, birthweight, axial length, in utero exposure to alcohol, and current smoking and alcohol consumption (Lee, Mackey, et al., 2021).

* Significant at two‐sided *p* < 0.05.

### 3.2 Childhood smoking exposure

#### 3.2.1 COPSAC2000

The most prominent postnatal exposure to smoking was in 20 participants who, in addition to prenatal and childhood passive smoking, also actively smoked at age 18 years. In the unadjusted analysis, significant deficits were found in the nasal (−9.8%; −17.8 to −1.8%; *p* = 0.02) and inferior sector (−6.9%; −13.4 to −0.4%; *p* = 0.04). When adjusted, significant deficits were found in all sectors, peaking at −11.6% (−20.3 to −2.9%, *p* = 0.01) in the nasal sector and −11.0% (−17.1 to −4.9%, *p* < 0.001) in the adjacent inferior sector (Table 5).

**TABLE 5:** Different amounts of exposure to postnatal smoking and associations with retinal nerve fibre layer thickness in young adults aged 18 years (COPSAC2000).

RNFL sector	RNFL thickness, mean (SD), μm, no smoking exposure (*n* = 91)	Smoking group A vs. no smoking exposure, mean absolute difference (95% CI)^a^, μm (*n* = 30)	*p‐*Value	Smoking group B vs. no smoking exposure, mean absolute difference (95% CI)^a^, μm (*n* = 20)	Smoking group B vs. no smoking exposure, mean relative difference (95% CI)^a^, % (*n* = 20)	*p*‐Value
Global	97.5 (9.7)	−9.6 (−13.4 to −5.8)	< 0.0001*	−9.1 (−13.6 to −4.6)	−9.3 (−13.9 to −4.7)	0.0001*
Superior	125.5 (15.3)	−12.8 (−19.3 to −6.3)	0.0002*	−9.7 (−17.4 to −2.0)	−7.7 (−13.9 to −1.6)	0.02*
Nasal	71.4 (11.4)	−3.2 (−8.4 to 2.0)	0.24	−8.3 (−14.5 to −2.1)	−11.6 (−20.3 to −2.9)	0.01*
Inferior	127.8 (18.0)	−17.7 (−24.2 to −11.2)	<0.0001*	−14.0 (−21.8 to −6.2)	−11.0 (−17.1 to −4.9)	0.0005*
Temporal	65.6 (8.3)	−5.0 (−8.9 to −1.1)	0.01*	−4.9 (−9.5 to −0.3)	−7.5 (−14.5 to −0.005)	0.04*

*Note*: Smoking group A (Zhu et al., 2023): Exposure to smoking during pregnancy and passive smoking during childhood but no exposure to smoking at age 18 years. Smoking group B (Zhu et al., 2023): Exposure to smoking during pregnancy and passive smoking during childhood plus exposure to smoking at age 18 years.

^a^ Adjusted for age, sex, birthweight, axial length, maternal alcohol use, alcohol use at 18 years and inhaled and/or intranasal corticosteroid use.

* Significant at two‐sided *p* < 0.05.

### 3.3 Combined meta‐analysis of all three cohorts

**FIGURE 1:**
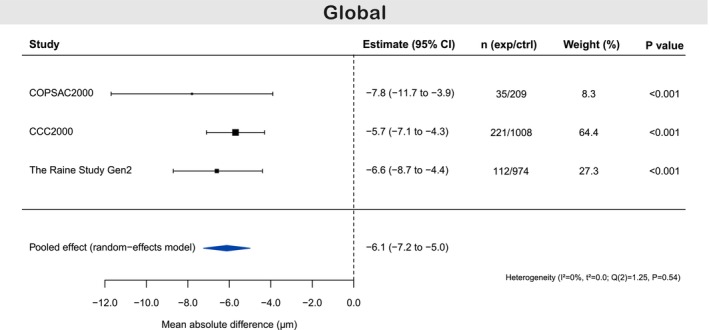
Forest plot of the associations between exposure to prenatal smoking and global RNFL thickness for all three cohorts in a meta‐analysis. RNFL, retinal nerve fibre layer. Exp, exposed. Ctrl, control.

**FIGURE 2:**
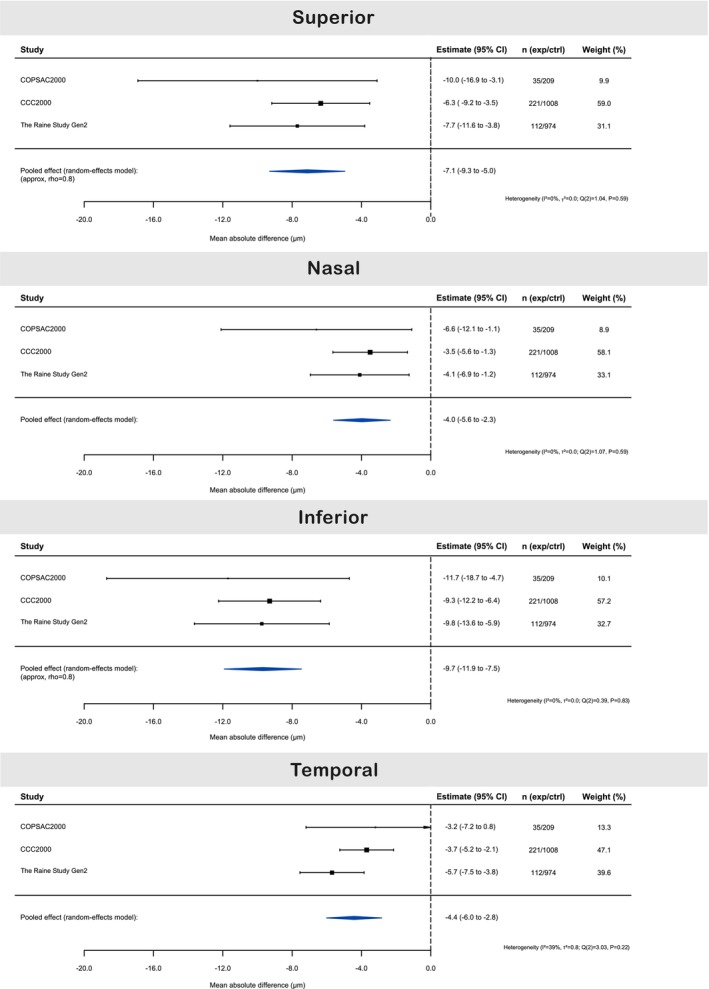
Forest plots of the associations between exposure to prenatal smoking and sectoral RNFL thickness for all three cohorts in meta‐analyses. RNFL, retinal nerve fibre layer.

We performed a combined meta‐analysis of the COPSAC2000, CCC2000 and The Raine Study Gen2 of both the global and the different RNFL sectors. Prenatal smoking was associated with a deficit in the global RNFL thickness in a combined meta‐analysis of the three cohorts; mean absolute difference −6.1 μm (−7.2 to −5.0) (Figure 1). This was similar for all sectors, showing decreased RNFL thickness from exposure to prenatal smoking: superior −7.1 μm (−9.3 to −5.0), nasal −4.0 μm (−5.6 to −2.3), inferior −9.7 μm (−11.9 to −7.5) and temporal −4.4 μm (−6.0 to −2.8) (Figure 2).

## 4 DISCUSSION

This combined meta‐analysis of three independent birth cohorts with expanded sectorial analyses utilizing data from three independent child cohorts found that not only was maternal smoking throughout pregnancy associated with a general deficit in peripapillary RNFL thickness, it also related to deficits in each sector (superior, inferior, nasal and temporal). The results are in line with our previous finding suggesting that the optic nerve is most vulnerable during prenatal life (Zhu et al., 2023).

Limitations of the study include variations between the cohorts in the number and type of study variables, especially the number of cigarettes per day, the associated recall bias and the degree to which tobacco smoke was inhaled. The smoking history was collected differently in the three cohort studies so a dosis effect of smoking exposure could not be calculated or compared between studies. Retinal and optic nerve fibres vary in individual thickness; thus, a RNFL thickness deficit is not necessarily a defect in the number of fibres in the optic nerve. It remains to be determined if the thickness deficit associated with prenatal tobacco smoke exposure is accompanied by a deficit in function of the central visual field measured by automated perimetry.

Studies have shown subnormal retinal blood flow in relation to smoking (Morgado et al., 1994; Steigerwalt Jr et al., 2000) and subnormal retinal vessel density in smokers, which may be related to the exposure to nicotine (Aboud et al., 2002). Tobacco smoke also contains oxidizing molecules that can lead to free radical production, oxidative stress and cellular damage (Bertram et al., 2009).

Hereditary optic neuropathy is predominantly associated with mitochondrial genetic defects, and toxic optic neuropathy is associated with poisons that inhibit cellular respiration, and with medications that block the function of bacterial ribosomes and possibly also the ribosomes of human mitochondria. A toxic effect on mitochondrial function of components of tobacco smoke that reach the fetus is also suspected (Fetterman et al., 2017).

The smoking‐related RNFL pattern of an inferior and nasal peripapillary deficit differs from the inferotemporal defect typical of autosomal‐dominant optic atrophy and the temporal papillomacular defect in Leber hereditary optic atrophy (Wang et al., 2021) (Park & Hwang, 2015). This information may assist clinical diagnosis and the study of optic neuropathy. Glaucoma is a progressing optic neuropathy and a leading cause of blindness worldwide. Common risk factors include advancing age, elevated intraocular pressure, family history and ethnicity. Early detection is crucial since early treatment can slow disease progression (Wagner et al., 2022). Assessment of the vertical cup/disc ratio and the RNFL are important parts of glaucoma screening (Larrosa et al., 2015). In eyes with glaucoma, the greatest RNFL deficit is seen in the inferior sector (Bowd et al., 2000; Larrosa et al., 2015). Our combined meta‐analysis found that the smoking‐related deficits in the peripapillary RNFL were most prominent in the inferior and superior sectors. This finding suggests that exposure to tobacco smoke might predispose to or aggravate the effects of glaucoma later in life.

To protect offspring from smoking‐related retinal nerve fibre damage, women considering pregnancy or already pregnant must be strongly counselled to quit smoking and offered support as well as nicotine replacement therapy if necessary. Knowing that susceptibility to optic nerve disease such as glaucoma is related to one's nerve fibre reserve, which shrinks with age and cannot be replenished, may be valuable messaging for encouraging women to quit for their child's eye health.

This combined meta‐analysis of three independent birth cohorts suggests that prenatal smoking exposure was related to overall deficits in the RNFL thickness. The largest deficits in the inferior and nasal sectors might reveal patterns that may assist in differential diagnosis and elucidation of eye disease mechanisms.

## AUTHOR CONTRIBUTIONS

LZ has written the first draft of the manuscript. All co‐authors have provided important intellectual input and contributed considerably to the analyses and interpretation of the data. All authors guarantee that the accuracy and integrity of any part of the work have been appropriately investigated and resolved and all have approved the final version of the manuscript. The corresponding author had full access to the data and had final responsibility for the decision to submit for publication.

## FUNDING INFORMATION

All funding received by COPSAC is listed on www.copsac.com. The Lundbeck Foundation (Grant no. R16‐A1694); the Ministry of Health (Grant no. 903516), the Danish Council for Strategic Research (Grant no. 0603‐00280B) and the Capital Region Research Foundation have provided core support to the COPSAC research centre. This project has received funding from the European Research Council (ERC) under the European Union's Horizon 2020 research and innovation programme (grant agreement no. 946228) to B.C. and from NIH grant R01 HL129735 to K.B. The core management of the Raine Study is funded by The University of Western Australia, Curtin University, Telethon Kids Institute, Women and Infants Research Foundation, Edith Cowan University, Murdoch University, The University of Notre Dame Australia and the Raine Medical Research Foundation. The eye data collection of the Raine Study Gen2 20‐year follow‐up was funded by NHMRC Grant 1021105, Ophthalmic Research Institute of Australia (ORIA), Alcon Research Institute, Lions Eye Institute and the Australian Foundation for the Prevention of Blindness. D.M. is funded by a NHMRC Practitioner Fellowship. The RNFL analysis is funded by an NHMRC Program grant. No honorarium, grant or other form of payment was given to any of the authors to produce this manuscript.

## CONFLICT OF INTEREST STATEMENT

DM consults for Novartis. The other authors declare no potential, perceived or real conflict of interest regarding the content of this manuscript. The funding agencies did not have any role in design and conduct of the study; collection, management and interpretation of the data; or preparation, review or approval of the manuscript. No pharmaceutical or other commercial company was involved in the study.

## ETHICS STATEMENT

We are aware of and comply with recognized codes of good research practice, including the Danish Code of Conduct for Research Integrity. We comply with national and international rules on the safety and rights of patients and healthy subjects, including Good Clinical Practice (GCP) as defined in the EU's Directive on Good Clinical Practice, the International Conference on Harmonization's (ICH) good clinical practice guidelines and the Helsinki Declaration. We follow national and international rules on the processing of personal data, including the Danish Act on Processing of Personal Data and the practice of the Danish Data Inspectorate.
